# Dreaming of being chased reflects waking-life experiences related to negative relationships with others metaphorically

**DOI:** 10.3389/fpsyg.2024.1413011

**Published:** 2024-07-26

**Authors:** Jiaxi Wang, Xiaoling Feng

**Affiliations:** ^1^School of Psychology, Guangzhou University, Guangzhou, China; ^2^Research Center for Embodied Cognition, Department of Psychology, School of Education, Guangzhou University, Guangzhou, China; ^3^Institute of Analytical Psychology, City University of Macau, Macau, China

**Keywords:** content analysis, continuity hypothesis, day-residue effect, dreams, Freud, metaphor, threat simulation theory, typical dreams

## Abstract

**Introduction:**

It has long been argued that there are dream metaphors which express waking-life experiences indirectly. Most of empirical evidence concerning this topic was in a qualitative way, while few studies explored the topic in a quantitative way. Under this background, we investigated whether dreaming of the typical theme ‘being chased or pursued’ was a metaphorical expression for waking-life experiences related to ‘negative relationships with others’.

**Methods:**

One hundred and sixty participants reported their waking-life experiences and dreams for a single day. Following this, two external judges rated whether there were any elements related to ‘negative relationships with others’ in both waking-life experiences and dreams. In addition, the judges assessed if there was any content related to ‘being chased or pursued’ in both waking-life experiences and dreams.

**Results:**

The frequency of dreaming of ‘negative relationships with others’ was higher than the frequency of the same topic in waking-life experiences, which in turn was higher than the frequency of typical theme dreaming of ‘being chased or pursued’. In addition, ‘negative relationships with others’ in waking-life experiences were correlated with both dreaming of ‘being chased or pursued’, and ‘negative relationships with others’ in dreams.

**Conclusion:**

These results suggested that the typical theme ‘being chased or pursued’ in dreams may represent some waking-life experiences metaphorically. In addition, the results support the threat simulation theory of dreaming, which suggests that threatening events in waking life increase the possibility of threatening events in dreams.

## 1 Introduction

Dreams are mysterious subjective experiences for many people. Freud (1900) indicates that waking-life experiences can be incorporated into dreams of the same day. This is termed as the day-residue effect. Hall and Nordby (1972) suggests that some waking-life experiences may appear in dreams directly, while others may be incorporated into dreams indirectly. The latter situation is related to the existence of dream metaphors, which express waking-life experiences metaphorically (Lakoff, 1993; Hartmann, 2011; Malinowski and Horton, 2015). The existence of dream metaphors may increase the difficulty to associate waking-life experiences with dreams, because sometimes there may be only emotional continuity between dreams and waking life (e.g., Hartmann, 2011; Schredl, 2012). For example, a person may dream about flying in the sky without any aid (e.g., the use of plane). The situation is not similar with waking life, because human can not fly. So it may be hard to associate the flying dream with waking life. From the perspective of dream metaphors, the dreamer may encounter a daily event which causes a feeling of freedom, while in the flying dream the dreamer also had a feeling of freedom. Thus there may be an emotional continuity between the daily event and the flying dream. So the flying dream may express the daily event indirectly.

Empirical evidence suggests that the frequency of an element in a dream series may predict the possibility of the intensity of a waking concern toward the element (e.g., Domhoff, 2017, 2020). So the day-residue effect may be affected by the intensity of waking concerns. Some findings supported the idea (Cartwright et al., 1969; Nikles et al., 1998; Cipolli et al., 2004; Cartwright et al., 2006; Schredl, 2006; Bradshaw et al., 2016; Wang et al., 2022a,b), while others did not (Foulkes and Rechtschaffen, 1964; Griffin and Foulkes, 1977; Wegner et al., 2004). One possible reason for the inconsistency may be the existence of dream metaphors, which may cause the floor-effect for some studies, because sometimes there may be only emotional continuity between dreams and waking life. Under this background, some studies turned their attention to showing that the intensity of waking concerns affected dream emotions. For example, watching stressful films enhanced the anxious emotions in dreams (Goodenough et al., 1975; Lauer et al., 1987), and presleep suggestion related to a phobic object affected the valence of dream emotions (De Koninck and Brunette, 1991). These results suggested that dreams offered benefits for processing emotions of waking-life experiences (e.g., Malinowski and Horton, 2015). To our knowledge, there was a lack of research studying dream metaphors quantitatively. Here we made some efforts for this topic.

Typical dreams are defined as dreams with similar contents reported by a high percentage of dreamers (Mathes et al., 2014). Previous studies indicated that different typical themes have different possibility to appear in dreams (e.g., Maggiolini et al., 2010; Mathes et al., 2014; Yu, 2015). Generally the rank of possibility of typical themes in dreams is stable over different sample populations, with some exceptions (Mathes et al., 2014). For example, on the one hand, some themes, such as ‘being chased or pursued’, ‘school, teachers, and studying’, and ‘eating delicious foods’ happen frequently in both German studies (e.g., Mathes et al., 2014), and Chinese studies (e.g., Yu, 2015). On the other hand, some theme, such as ‘sexual experiences’, was ranked relatively highly in the German studies (Mathes et al., 2014), but not for Chinese samples (Yu, 2015). The potential differences in the rank of frequency of typical themes in dreams may be due to dream metaphors. For example, Mathes et al. (2014) found that women dreamed more often about ‘Floods or tidal waves’, ‘Swimming’ than men, which was explained by the metaphorical meaning of typical themes in dreams, such that flood in dreams may reflect the feeling of ‘overwhelmed’ in waking life.

The typical theme ‘being chased or pursued’ happens frequently in dreams (Mathes et al., 2014; Yu, 2015). Yet in waking life this situation may not happen frequently, because this kind of behavior is not permitted by laws. Typically there are waking-life experiences related to negative relationships with others. As the day-residue may be affected by the intensity of waking concerns, some waking concerns for negative social situations may be related to dreaming of the theme ‘being chased or pursued’. In addition to the theme ‘being chased or pursued’ in dreams, there are also other kinds of ways that describe social negative situations, such as verbal aggression, some of which may also be affected by waking concerns for negative social situations. Here we hypothesized that ‘negative relationships with others’ in waking life were correlated with both dreaming of the typical theme ‘being chased or pursued’ and dreaming of ‘negative relationships with others’.

## 2 Methods

### 2.1 Participants

Originally two hundred and nine participants participated in this study, but forty nine participants failed to report their dreams. As a result, the data of one hundred and sixty participants [23 males, 137 females; mean (SD, range) age 21.11 (2.37, 18–31) years] was used for further analyses. They were college students in China, and they were self-reported to be sufficient with the following criteria: recall at least three dreams per week; sleep at least 6 h per night; take no more than 30 min to fall asleep; have no neurological or psychiatric history. All subjects gave written informed consent before the start of the study, and the study was approved by the Research Ethics Committee of the local university.

### 2.2 Material

#### 2.2.1 Daily diary

The method to collect daily diary was similar to van Rijn et al. (2015). Up to three items could be recorded in each category: *1. Major Daily Activities (MDAs): activities that took up most of the participants’ time during the day,* e.g.*, going to work or university, meals, shopping. 2. Personally Significant Experiences (PSEs): important daily events that may or may not have taken up much time,* e.g.*, emotional events. 3. Major Concerns (MCs): concerns or thoughts that participants had on their mind during the day that may not have taken up much time, but were still considered important to them,* e.g.*, money problems, examination stress.*

In addition, participants rated the emotionality of each waking-life experience, by a 5-point Likert score.

#### 2.2.2 Dream report

The method of recording a dream diary was the following: *Describe everything in your dreams, with as much detail as possible: what happened, in what time frame, with whom,* etc. *Describe the cognitions, emotions, and behaviors you experienced in your dream, as well as the cognitions, emotions, and behaviors of all other parties included in your dream (if evident to you). If it was a lucid dream, state so.*

#### 2.2.3 Content analysis and data analysis

In this study, ‘negative relationships with others’ during both waking-life experiences and dreams were rated by the ‘negative social interaction events’ category of the scale in Wang et al. (2021), which contained the following six categories: ‘physical violence’, ‘verbal aggression’, ‘forcing’, ‘unconsentful sexual interaction’, ‘avoidence behavior’, and ‘abandonment’. In addition, ‘being chased or pursued’ during both waking-life experiences and dreams were rated. Note that each participant’s night dream and daytime waking-life experience were only rated once, regardless of the number of night dreams and the number of waking-life experiences. In other words, each participant only provided one data for ‘negative relationships with others’ during waking-life experience, ‘negative relationships with others’ during dreams, ‘being chased or pursued’ during waking-life experiences, and ‘being chased or pursued’ during dreams, separately.

A score of “0” for no presence of target content or “1” for presence of target content was used for analysis. McNemar tests were used to analyze potential differences betwen different variables, and phicoefficient tests were used to analyze potential correlations between different variables.

### 2.3 Procedure

Recruitment was conducted via online wechat groups which were created for recruiting participants who wanted to take part in psychological experiements. These participants were students of different universities in GuangZhou city, such as the South China Normal University and the Guangzhou University. As a reward, they could get feedback about their dream reports and a little money. Participants recorded their dreams and waking-life experiences for one day in a spreadsheet at home via an online web. Specifically, in the evening participants recorded three kinds of waking-life experiences, and rated the emotionality of each waking-life experience, by a 5-point Likert scale. In the next morning participants reported their last night dreams immediately when they got up. Then two blind external judges rated if there was any content related to ‘negative relationships with others’ in both waking-life experiences and dreams, according to the following six categories: ‘physical violence’, ‘verbal aggression’, ‘forcing’, ‘unconsentful sexual interaction’, ‘avoidence behavior’, and ‘abandonment’. In addition, the judges rated if there was any content related to the theme ‘being chased or pursued’ in both daily diaries and dreams. A score of “0” for no presence of target content or “1” for presence of target content was used. The Cronbach’s consistencies coefficient (α) among the two judges was from 0.78 (the variable ‘negative relationships with others’ in dreams) to 1 (the variable ‘being chased or pursued’ in waking-life experiences). Inconsistent ratings were discussed later until an agreement was reached. All statistical analyses were done by SPSS software.

## 3 Results

The total number of dreams was 192. Two lucid dreams were reported. The average length of dreams was 149.6 (SD = 133.2, range from 16 to 818) words. The mean emotionality of MDAs was 3.46 (SD = 0.72), and the mean emotionality of PSEs was 4.1 (SD = 0.77), and the mean emotionality of MCs was 4.18 (SD = 0.70). In the following, all number of frequency represented the number of the participant who reported relevant content, because each participant (N = 160) only provided one data for the following variables (detail see section 2.2.3). There was no recording of waking-life experiences related to ‘being chased or pursued’. The frequency of dreaming of ‘being chased or pursued’ was 11.9% (*N* = 19). The frequency of waking-life experiences related to ‘negative relationships with others’ was 20% (*N* = 32). The frequency of dreaming of ‘negative relationships with others’ was 34.4% (*N* = 55). Among the results, 20 participants reported ‘negative relationship with others’ during both waking-life experiences and dreams, and 11 participants reported ‘negative relationship with others’ during waking-life experiences and ‘being chased or pursued’ during dreams.

McNemar tests showed that the frequency of dreaming of ‘negative relationship with others’ was higher than the frequency of waking-life experiences related to ‘negative relationships with others’ (*χ*^2^ = 10.30, *p* = 0.001), and the latter kind of recording was higher than the frequency of dreaming of ‘being chased or pursued’ (*χ*^2^ = 4.97, *p* = 0.026). These results suggested that ‘negative relationships with others’ in dreams happened more frequently than in waking life, which in turn happened more frequently than the typical theme ‘being chased or pursued’ in dreams.

In addition, phicoefficient tests suggested that the frequency of ‘negative relationships with others’ during waking-life experiences was correlated with both dreaming of ‘being chased or pursued’ (phi = 0.35, *p* < 0.001), and dreaming of ‘negative relationships with others’ (phi = 0.30, *p* < 0.001). In addition, the latter two kinds of recordings were significantly correlated with each other (phi = 0.34, *p* < 0.001).

## 4 Discussion

Our results showed that 11.9% of participants dreamed of ‘being chased or pursued’. The number was similar to Mathes et al. (2014) where the frequency for the typical theme was 11.04%. By contrast, Yu (2015) only found 4.8% of diary dreams were related to the theme. Yu (2015) noted that in their study judges were not familiar with the rating system for typical themes in dreams, so they may underestimate the incidence of the themes. Here we found a higher frequency of dreaming of the theme ‘being chased or pursued’ than Yu (2015), which may be in line with Yu’s (2015) idea that the frequency of dreaming of ‘being chased or pursued’ in their study was underestimated.

We confirmed our hypothesis by the result that ‘negative relationships with others’ in waking life was correlated with the frequency of dreaming of ‘being chased or pursued’. By contrast, participants did not report any waking-life experiences related to the theme ‘being chased or pursued’. In addition, the frequency of dreaming of ‘being chased or pursued’ was lower than the frequency of ‘negative relationships with others’ in waking-life experiences. As the intensity of waking concerns may affect the possibility of dreaming of the concerns metaphorically (Schredl, 2012), these results suggested that the typical theme ‘being chased or pursued’ in dreams may represent some waking-life experiences metaphorically. Here our results may provide some quantitative evidence for this topic, because they may indicate that some typical themes in dreams were metaphorical expressions for waking-life experiences.

In addition, our result suggested that 34.4% of dreams were related to ‘negative relationships with others’, which was similar to Wang et al. (2021) where the frequency was 37.4%. We confirmed another hypothesis by the finding that ‘negative relationships with others’ in waking life was correlated with dreaming of the same topic. This result support the threat simulation theory of dreaming which suggests that threatening events in waking life increased the possibility of dreaming of threatening events in dreams (Revonsuo, 2000). In addition, we found that the frequency of ‘negative relationships with others’ in waking life was lower than the frequency of dreaming of the same topic. This result also supported the threat simulation theory, because the theory predicts that the frequency of dreaming of threatening events can be higher than the frequency of threatening events in waking life.

Concerning the topic of the continuity between waking-life experiences and dreams, some studies used a similar paradigm for the purpose. In these studies participants recorded their waking-life experiences and dreams at first. Then both participants and judges rated if there are similar elements (e.g., characters, objects, actions, locations etc.) between the two kinds of recordings [for an introduction, see Wang et al. (2020)]. Similar elements may be defined as having a semantically associative relationship between different kinds of memories. Two elements which belonged to a same category may be rated as similar elements [e.g., father and mother, detail see Appendix in Wang et al. (2023)]. A problem was that some dream metaphors may not have semantic relationship with waking-life experiences which were related to the metaphors indirectly. For example, in this study we found that the theme “being chased or pursued” was a metaphor for waking-life experiences related to negative relationships with others. As stated in the introduction section, some studies found that the intensity of waking-life experiences affected dreams, while others did not. Our result may imply that the reason for the latter studies was because the existence of dream metaphors enhanced the difficulty to find potential correlations between target stimuli and dreams, which caused a floor-effect.

### 4.1 Limitation

Firstly, most of our participants were females, because in the online wechat group recruiting participants there were more female students than male students, and female students may be more interested in dream studies than male students, so it was not clear if our finding here was suitable for males. However, as previous studies did not find differences in dreaming of “being chased or pursued” between men and women (e.g., Mathes et al., 2014), this limitation may not affect our result. Nevertheless, future studies used a balanced sex sample are welcomed. In addition, in this study, another limitation caused by participants was that all inclusion/exclusion criteria were via self-reported questions, as opposed to accurate scales. The criteria adopted here was similar to the criteria of van Rijn et al. (2015), which provided the paradigm to record waking-life experiences for this study. Future study should improve this limitation by using a more accurate scale, such as the Pittsburgh sleep quality index (PSQI).

Secondly, although we found there was a correlation between waking-life experiences related to negative relationships with others and dreaming of “being chased or pursued,” we did not ask participants to report whether the two kinds of memories were related with each other. So we did not know if participants agreed that the latter kind of dream represented the former kind of waking-life experiences metaphorically. Future research can use a qualitative way to test this idea.

Thirdly, in this study, we only explored whether the ‘negative relationships with others’ during waking-life experiences was related to dreaming of ‘being chased or pursued’, so it was unclear if other kind of negative waking-life experience was also related to this kind of dream. As stated above, sometimes there is only emotional continuity between waking-life experiences and dreams. According to the continuity hypothesis of dreaming, there is cognitive continuity between dreaming and waking life (e.g., Schredl, 2012). Both the ‘being chased or pursued’ situation and the ‘negative relationships with others’ situation were related to characters, while other kind of negative situation, such as flood, may not be related to characters. As emotions are related to cognitive appraisal of situations, appraisals of the former two situations may be more similar than the appraisal of the other situation, which in turn increased the similarity in emotions between the former two situations than the other situation. Given that our purpose is to explore dream metaphors quantitatively, we chose the most likely situation for our purpose. Future research can explore the correlation between other kind of negative waking-life experience and dreaming of ‘being chased or pursued’.

## Data availability statement

The data that supports the findings of this study are available on request from the corresponding author upon reasonable request.

## Ethics statement

The studies involving humans were approved by the Ethics Committee of Guangzhou University. The studies were conducted in accordance with the local legislation and institutional requirements. The participants provided their written informed consent to participate in this study.

## Author contributions

JW: Conceptualization, Funding acquisition, Investigation, Methodology, Writing – original draft. XF: Formal analysis, Investigation, Resources, Software, Supervision, Validation, Writing – review & editing.
